# Multiplexed Near-Field Optical Trapping Exploiting Anapole States

**DOI:** 10.1021/acsnano.3c03100

**Published:** 2023-08-21

**Authors:** Donato Conteduca, Giuseppe Brunetti, Isabel Barth, Steven D. Quinn, Caterina Ciminelli, Thomas F. Krauss

**Affiliations:** † School of Physics, Engineering and Technology, University of York, Heslington, York YO10 5DD, United Kingdom; ‡ Optoelectronics Laboratory, Politecnico di Bari, 70125 Bari, Italy; § York Biomedical Research Institute, University of York, Heslington, York YO10 5DD, United Kingdom

**Keywords:** optical trapping, nanotweezers, anapole state, multiplexed trapping, dielectric metasurface, vesicles

## Abstract

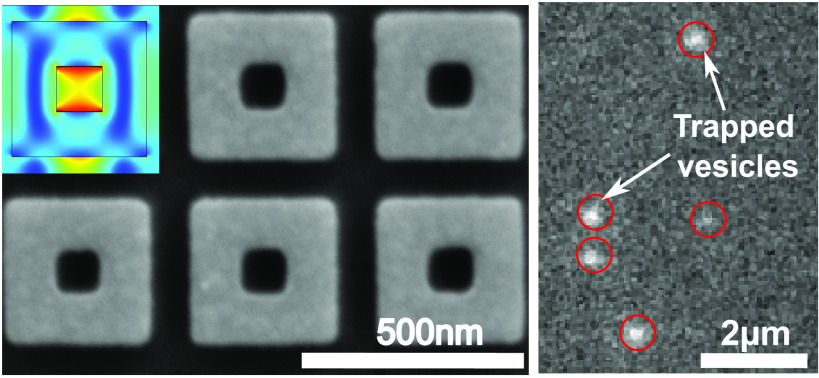

Optical tweezers have had a major impact on bioscience research by enabling the study of biological particles with high accuracy. The focus so far has been on trapping individual particles, ranging from the cellular to the molecular level. However, biology is intrinsically heterogeneous; therefore, access to variations within the same population and species is necessary for the rigorous understanding of a biological system. Optical tweezers have demonstrated the ability of trapping multiple targets in parallel; however, the multiplexing capability becomes a challenge when moving toward the nanoscale. Here, we experimentally demonstrate a resonant metasurface that is capable of trapping a high number of nanoparticles in parallel, thereby opening up the field to large-scale multiplexed optical trapping. The unit cell of the metasurface supports an anapole state that generates a strong field enhancement for low-power near-field trapping; importantly, the anapole state is also more angle-tolerant than comparable resonant modes, which allows its excitation with a focused light beam, necessary for generating the required power density and optical forces. We use the anapole state to demonstrate the trapping of 100’s of 100 nm polystyrene beads over a 10 min period, as well as the multiplexed trapping of lipid vesicles with a moderate intensity of <250 μW/μm^2^. This demonstration will enable studies relating to the heterogeneity of biological systems, such as viruses, extracellular vesicles, and other bioparticles at the nanoscale.

## Introduction

Optical tweezers have greatly advanced biological studies on cells and bacteria with the ability to investigate targets in a label-free and contact-free manner.^1,2^ Nanostructures can improve the trapping efficiency of such tweezers because they offer near-field enhancement, which results in higher optical forces due to stronger field gradients.^3−5^ The near-field enhancement provides the ability to trap nanoparticles, such as DNA and single proteins,^6^ which is otherwise extremely difficult, as the trapping force scales with the volume of the particle. Most of the work so far has focused on individual nanoparticles, while biological systems are intrinsically heterogeneous. The next frontier is therefore the ability to trap hundreds of particles in parallel in order to gain more information about the biological system, such as variability and the presence of possible mutations together with a response to therapies and drugs, in order to compile meaningful statistics.^7−9^

The idea of creating multiple traps has previously been put forward by using multiple lasers,^10^ by implementing acousto-optic beam deflectors (AODs),^11^ or by using spatial light modulators (SLMs).^12^ However, these methods are all based on far-field Gaussian beams that are best suited for trapping at the microscale due to their relatively weak gradient force, which can be orders of magnitude lower than that in the near field. Nanoplasmonic configurations, such as nanoantennas,^13,14^ have demonstrated trapping and manipulation of tens or more objects in parallel with higher stability, compared to bulk tweezers. A large number of multiple trapping events in parallel was observed with a minimum size of sub-micrometer (>500 nm) or even larger beads, a necessary condition to limit the power demand and to minimize the thermal effects.^14^ Moving toward the nanoscale, the control of thermal effects becomes a challenge. In this context, photonic nanojets have been used to trap tens of 190 nm polystyrene beads in parallel, as well as *Escherichia coli* bacteria.^15^ However, nanojets still require relatively high intensities of ∼1 mW/trap, because they do not provide gradient forces as high as nanotweezers realized with dielectric or plasmonic nanostructures. For example, the strong hotspots available with a plasmonic resonant metasurface have enabled the multiplexed trapping of dielectric nanobeads as small as 20 nm, i.e., 10 times smaller than the polystyrene particles mentioned above, yet with a lower intensity of ∼250 μW/trap.^16,17^ The main advantage is offered by the resonant behavior in the plasmonic array, which guarantees a stronger gradient field. The issue with such plasmonic nanostructures, however, is their intrinsic absorption loss, which causes significant heating and results in thermophoresis, opposing the trapping force. As a result, only small arrays with less than 10 trapping events in a limited time scale have been realized so far in order to keep the power budget manageable.^17^

**Figure 1:**
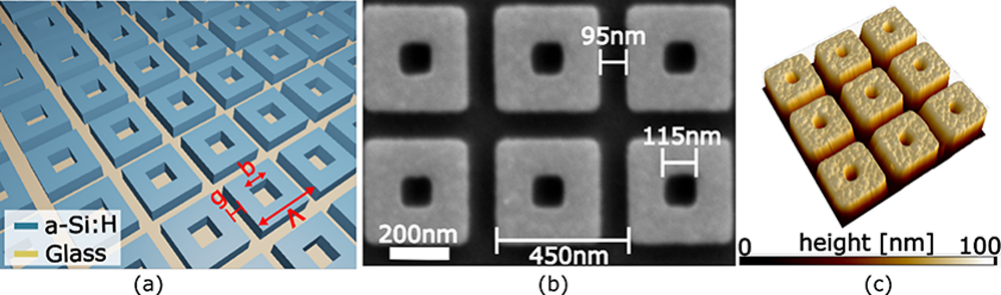
Dielectric metasurface with a nanocuboid array. (a) Schematic of the array. (b) SEM micrograph and (c) AFM image of a section of the dielectric metasurface.

Dielectric nanostructures offer a better compromise because, with their typical low optical losses, they minimize heating effects and maximize the energy confinement, thus achieving strong near-field gradient forces.^18−20^ The first demonstration of multiple trapping with dielectric configurations was achieved with silicon nanoantennas.^21^ Multiple nanoparticles of only 20 nm were trapped in each nanoantenna, due to the presence of many hotspots, thus creating multiple trapping sites. Negligible thermal effects were observed due to low absorption losses of silicon in the near-infrared wavelength region. However, the absence of a strong resonant behavior in the nanoantenna prevents achieving very strong energy confinement, requiring a power of a few milliwatts to excite each nanoantenna, which limits the total number of simultaneous trapping sites. Resonant dielectric configurations allow us to enhance the gradient field together with negligible thermal effects. The most advanced experimental work reported to date uses a nanohole array to achieve the multiplexed trapping of tens or more viruses, but still requiring a trapping power of around 1 mW/μm^2^, which needs a tightly focused beam and scanning of the beam for multiplexing.^19^ Here, we demonstrate a dielectric metasurface that provides a next-level multiplexing capability. The key to the scalability is our design, which exploits an anapole state that localizes the target particle in the center of a nanocuboid (Figure 1).^18^ Compared to our previous design in ref (18), in this work the choice of using a dielectric material with higher refractive index and low absorption losses at the operating trapping wavelength (λ ∼ 780 nm), e.g., hydrogenated amorphous silicon (a-Si:H), allows enhancement of the gradient field, achieving stronger trapping efficiency. Furthermore, this design has the advantage that the size of the nanocuboid can be tailored to the size of the target, hence exercising a degree of selectivity. More importantly, the anapole state, being highly localized in real space, exhibits a wide angular range in *k*-space, which allows excitation with a focused beam. This is highly advantageous for the trapping application and is in contrast to other resonant modes supported by metasurfaces that tend to be distributed in real space and are therefore highly sensitive to excitation angle.^22^ We have previously estimated, using numerical simulations, that an anapole-based metasurface can trap 100’s or even 1000’s of nanoparticles in parallel.^18^

We now experimentally validate the multiplexing capability of the anapole array by demonstrating the successful trapping of tens of 100 nm polystyrene particles in parallel, with a power density of only *I* ∼ 160 μW/μm^2^, with hundreds of beads trapped in only 10 min. In terms of biological particles, we have also observed the multiplexed trapping of unilamellar vesicles (∼100 nm), which represent an excellent model for viruses and exosomes.^23,24^ Such vesicles are susceptible to photodamage,^24^ which makes low-power trapping an essential requirement, which we meet by achieving stable trapping with *I* ∼ 250 μW/μm^2^.

## Results and Discussion

### Experimental Realization of the Anapole Metasurface

The metasurface is realized in a-Si:H. This material has a high refractive index (*n* = 3.6 at λ = 785 nm) and only minimal loss (*k* ∼ 10^–4^), which ensures that high-quality resonances can be obtained. We design the metasurface with a unit cell of the array consisting of a nanocuboid in a-Si:H with an inscribed hollow square. The gap between different cuboids is smaller than 100 nm, in order to guarantee that the trapping of the target objects (∼100 nm) can only occur in the hollow core of the cuboid.^18^ We used an a-Si:H layer of 100 nm thickness, deposited on a glass substrate (*n* = 1.46). Our design uses a period of *Λ* = 450 nm, a cuboid with a hole size of *b* = 115 nm, and a gap between the cuboids of *g* = 95 nm to provide a resonance condition at λ_R_ = 786 nm and the best compromise in terms of optical performance and reliable fabrication steps (Figure 1).^18,25,26^ The nanocuboids support an anapole state,^25,26^ created by the destructive interference between the electric and the toroidal radiation modes excited in the unit cell, which is responsible for the high *Q*-factor and the strong near-field confinement (see Supporting Information 1),^25^ which are fundamental for maximizing the trapping efficiency.

**Figure 2:**
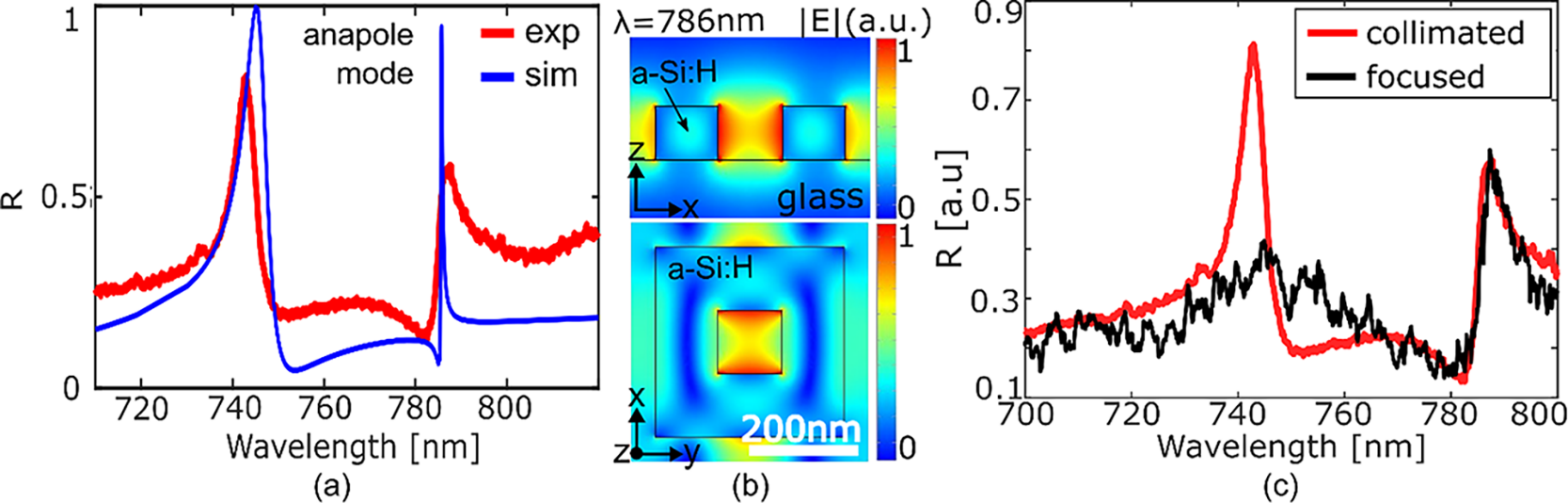
Numerical and experimental results of the anapole state. (a) Experimental (red curve) and simulated (blue curve) spectrum with collimated input light, supporting the coupled anapole state at λ_R_ = 786 nm. (b) Cross-section (top) and top view (bottom) of the optical confinement on resonance (3D FEM simulations). (c) Comparison of the experimental spectra with collimated (red curve) and focused (black curve) input light, clearly highlighting the angular tolerance of the anapole state. For the focused light, we have measured the spectrum with the same input condition used during the trapping experiments, while for collimated light the beam size is larger than 50 μm.

The resonance is excited with out-of-plane illumination by a laser beam with a relatively large diameter of 30 μm to allow the excitation of at least 3000 unit cells in parallel, thereby facilitating multiplexed optical trapping (see Experimental Section).^18^ The experimental results confirm that the coupled anapole state presents a relatively high *Q*-factor (*Q* ∼ 200) and a resonance amplitude *R* > 0.5 (Figure 2a). We attribute the mismatch of *Q*-factor and resonance amplitude between experiments and simulations (*Q*_sim_ ∼ 10^3^ and *R*_sim_ > 0.9) to fabrication tolerances and scattering losses (Figure 2a).

The numerical simulations based on the 3D finite element method (FEM) indicate a near-field enhancement *E*_ON-res_^2^/*E*_OFF-res_^2^ > 400 for the anapole state and tight field confinement (Supporting Information 2), both of which support efficient optical trapping (Figure 2b). We also note that in addition to the anapole state at λ_R_ = 786 nm, the spectrum in Figure 2a shows the presence of another resonant mode at λ = 745.4 nm (Supporting Information 3). This is a guided-mode resonance with the energy confined on the cuboid’s surface, which is spatially extended and therefore angularly very sensitive. The importance of the angular sensitivity is highlighted in Figure 2c. For collimated excitation, the distributed mode has a comparable *Q*-factor but is superior to the coupled anapole state in terms of the resonance amplitude (Figure 2a). However, for excitation with a focused beam, which is required for the trapping application to achieve reasonable intensities, the coupled anapole state clearly dominates over the distributed mode (Figure 2c and Supporting Information 4).^27,28^

### Numerical Simulations of the Trapping System

The trapping strength can be enhanced by the self-induced back-action (SIBA) effect^29−31^ via red-detuning the input laser by approximately 1 nm from the resonance. When the particle enters the trap, the change in the refractive index red-shifts the resonance to align it with the laser wavelength, which reinforces the trapping action.

**Figure 3:**
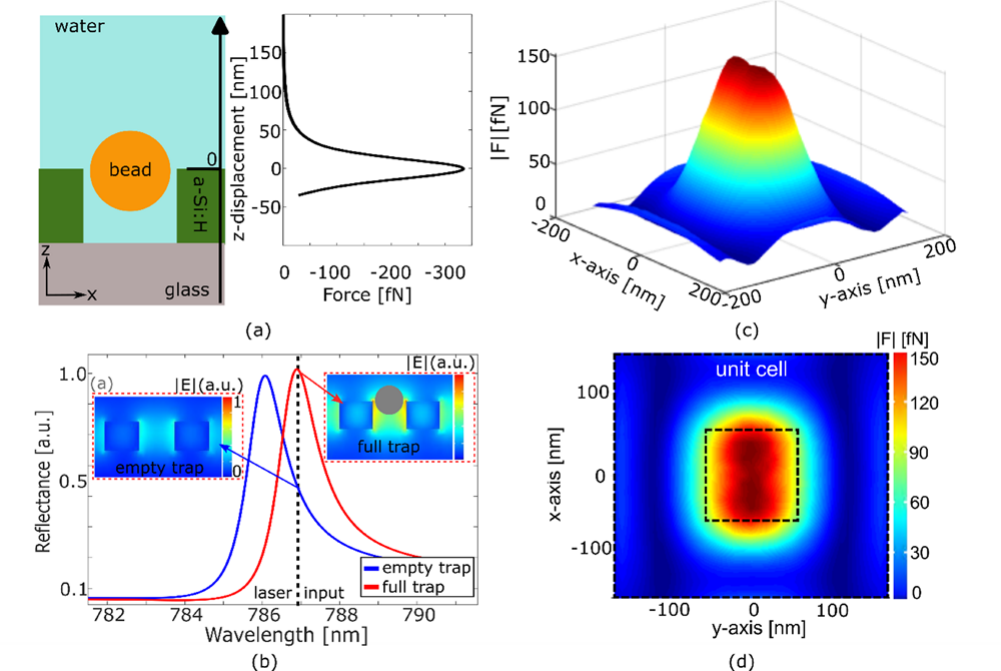
Trapping performance. (a) Schematic of the trapping system for a single unit cell of the metasurface (left) and trapping force calculated for different positions of the bead from the trapping site along the vertical direction (*z*-axis, with *x* = *y* = 0) with *I* = 100 μW/μm^2^. Numerical results confirm an optical stability of *S* = 10 with *I* = 300 μW/μm^2^ (Supporting Information 7). (b) Simulated resonance shift in the presence of the particle trapping due to the SIBA effect. The laser input wavelength is set at λ_in_ = 787 nm. The simulations in the insets show the difference in field distribution between an empty and a filled trapping site, illustrating the enhancement due to the SIBA effect. (c) Force distribution along the *x*- and *y*-axes within a unit cell of the array exerted on a 100 nm bead (*n* = 1.45) placed 50 nm above the cuboid surface and (d) top view in the inset.

The quality factor of the resonance of *Q* ∼ 200 ensures the effectiveness of the SIBA effect because the line width is larger than the 1 nm wavelength detuning observed when the particle enters the trapping site^32^ (Figure 3b).

We conducted 3D FEM simulations and applied the Maxwell stress tensor (MST) method^33^ to calculate the optical forces exerted by the anapole state on a 100 nm particle with a refractive index *n* ∼ 1.45, representative of vesicles or viruses, immersed in water (*n* = 1.33)^34^ (Figure 3a,c,d and Supporting Information 5). We aim for an optical stability of *S* = *U*/(*k*_B_·*T*) ≥ 1 (*U* is the potential energy required to bring the nanoparticle from a free position to the trapping site, *k*_B_ is the Boltzmann constant, and *T* is the temperature in Kelvin), to minimize the power requirement while ensuring that a trapping time of a few seconds can be achieved, which is sufficient for the characterization of the targets.^35^ The trapping stability is calculated by integrating the values of the optical forces as a function of particle displacement from the trapping site (Figure 3a), as described in detail in ref (34).

The large area of the array is also advantageous for initiating trapping, because a large number of sites increases the probability of a trapping event occurring spontaneously. Furthermore, it offers further trapping sites when the object is released from the initial site, in a “hopping” operation mode.^19,21^ “Hopping” increases the residence time for particles within the array, even for a low-power operation.

We calculate an optical power requirement of *I* = 30 μW/μm^2^ to achieve *S* ≥ 1, which corresponds to a total power of *P*_tot_ = 21 mW for a beam diameter of 30 μm.

### Experimental Demonstration of Multiplexed Trapping

**Figure 4:**
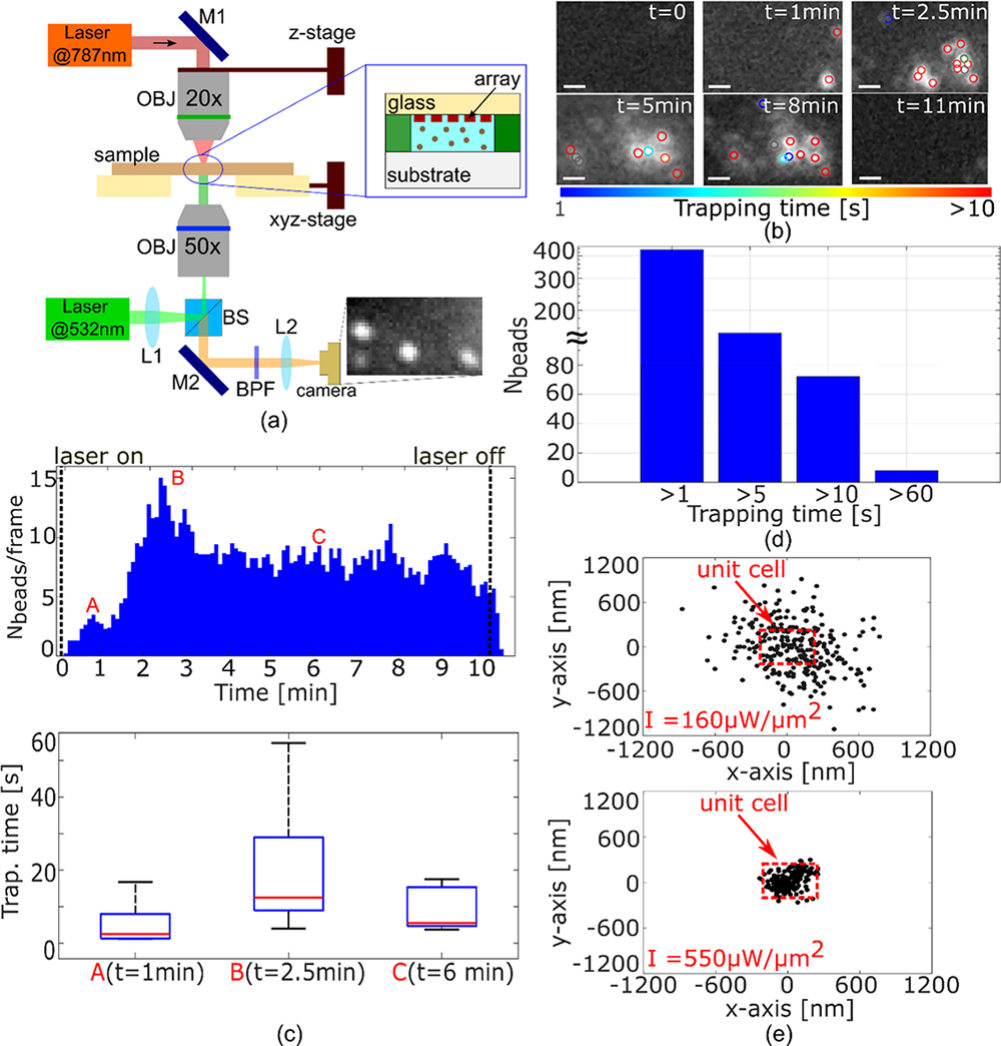
Experimental trapping of multiple 100 nm beads. (a) Schematic of the optical setup for multiplexed trapping at λ = 787 nm with integrated fluorescence excitation at λ = 532 nm. The trapping chamber is shown in the inset. Mi = mirrors, Li = optical lenses, BS = beam splitter, BPF = band-pass filter, OBJ = objective. (b) Time evolution of trapping of 100 nm polystyrene beads for the intensity of the trapping laser of *I* = 160 μW/μm^2^. Colored circles represent particles that are trapped for at least 1 s. The scale bar is 1.5 μm. (c) Number of beads trapped in parallel during an interval of *t* = 10 min (top) and average trapping time in different trapping conditions (bottom). The red lines in the box plots represent the median values of the displacement; the blue box and the black lines represent the 25th and 75th percentiles and the extreme values of the trapping time. (d) Total number of beads (*N*_beads_) trapped for *t* > 1 s, *t* > 5 s, *t* > 10 s, and *t* > 60 s. A linear scale is used for the *y*-axis for *N*_beads_ < 100, and a logarithmic scale for *N*_beads_ > 100. (e) Distribution of the position of a single particle trapped for *t* > 10 s with *I* = 160 μW/μm^2^ (top) and *I* = 550 μW/μm^2^ (bottom).

We initially used 100 nm polystyrene beads (*n* = 1.57) to verify the multiplexed trapping capability; the beads contain fluorophores for ease of tracking. The optical setup is described in Figure 4a, showing how the array is mounted in a microfluidic channel and facing downward. The focused trapping laser (RLS/785NM-600MW) is incident from the top (λ = 787 nm) and creates a 30 μm diameter spot. The metasurface is resonant at 786 nm, i.e., slightly detuned (∼1 nm) to exploit the SIBA effect (see Experimental Section). A second laser at 532 nm with *P* = 1 mW (Ventus 532), used to excite the fluorophores, is incident from below. The fluorescence signal (560 nm < λ_em_ < 590 nm) is then collected with a CCD camera. We observe multiple trapping events at an intensity of 160 μW/μm^2^ (Figure 4b). The surface is functionalized to prevent the beads from sticking to it even when the trapping laser is off (see Experimental Section).

We conducted 10 min long measurements with constant laser power in order to collect meaningful statistics. Note that this long time scale is only possible because of our use of low-loss dielectric materials.^17^ It takes only a few seconds for the first particle to be trapped spontaneously, because of the large size of the array. In total, we observe around 400 trapping events in *t* = 10 min, assuming a minimum trapping duration of *t*_trap_ ≥ 1 s, which would, for example, be sufficient to characterize a target with spectroscopic techniques.^36−38^ Many particles are trapped for longer times; for example, ∼100 beads show a trapping time of *t*_trap_ > 5 s (Figure 4b and d, Supporting Movie 1). Typically, we observe 10–15 beads to be trapped in parallel. For the trapping analysis, we identify each particle and track its position over time in the whole field of view in order to guarantee that each trapped bead is counted only once (see Experimental Section).

We are also able to confirm the SIBA effect experimentally. While particles are initially trapped in random locations across the array, they form a denser distribution within 1–2 min from laser activation (Figure 4b). We explain this effect with the local change of the resonance condition due to the presence of trapped particles, which increases the trapping force and attracts further particles. Within 2 min, we observe 10–15 particles to be trapped in a localized area (Figure 4b and c). After *t* = 2.5 min the number of beads trapped in parallel slightly decreases, which we associate with a redistribution of the particles that are trapped in close proximity in the array. Then, the number stabilizes until the laser is turned off (*t* = 10 min), with a full release of the beads (Figure 4b, Supporting Movie 2), confirming the purely optical nature of the trapping mechanism. The time that each particle spends inside a trap confirms this observation (Figure 4c); we note that the longest trapping time occurs when most particles are trapped in close proximity. Clearly, the anapole states interact across the array to generate a distributed SIBA effect that supports the formation of such high-density trapping areas.

We have also studied the mean displacement of the trapped beads as a function of input power (Figure 4e, Supporting Information 6). For an intensity of 160 μW/μm^2^ and for particles trapped for >10 s, they occupy an area up to 1 μm^2^, which corresponds to four unit cells, indicating that they can hop between neighboring cells. For an intensity of 550 μW/μm^2^, due to an increase of the optical stability with power (Supporting Information 6), they are more confined within a single unit cell of the array, yet there is still some evidence for hopping. One would expect that for even higher intensity, the particles could be localized perfectly; however, this is not what we observe. For an intensity of 800 μW/μm^2^, we instead observe a strong decrease in the localization of particles and evidence for thermophoresis. We calculate the corresponding temperature increase to be Δ*T* > 10 K, which is sufficient for overcoming the trapping action and for pushing particles away from the laser beam center into outer areas (Supporting Information 8).^39,40^ Regarding heating at lower power, we have also calculated the temperature change when illuminating the array with *I* = 160 μW/μm^2^ (Figure 4) and note a temperature increase, in water, of Δ*T* ∼ 2.9 K (see Supporting Information 9 for details). We note that this smaller temperature increase associated with low power operation is not sufficient to cause any detrimental thermal effects.^12^

### Trapping of Unilamellar Vesicles

**Figure 5:**
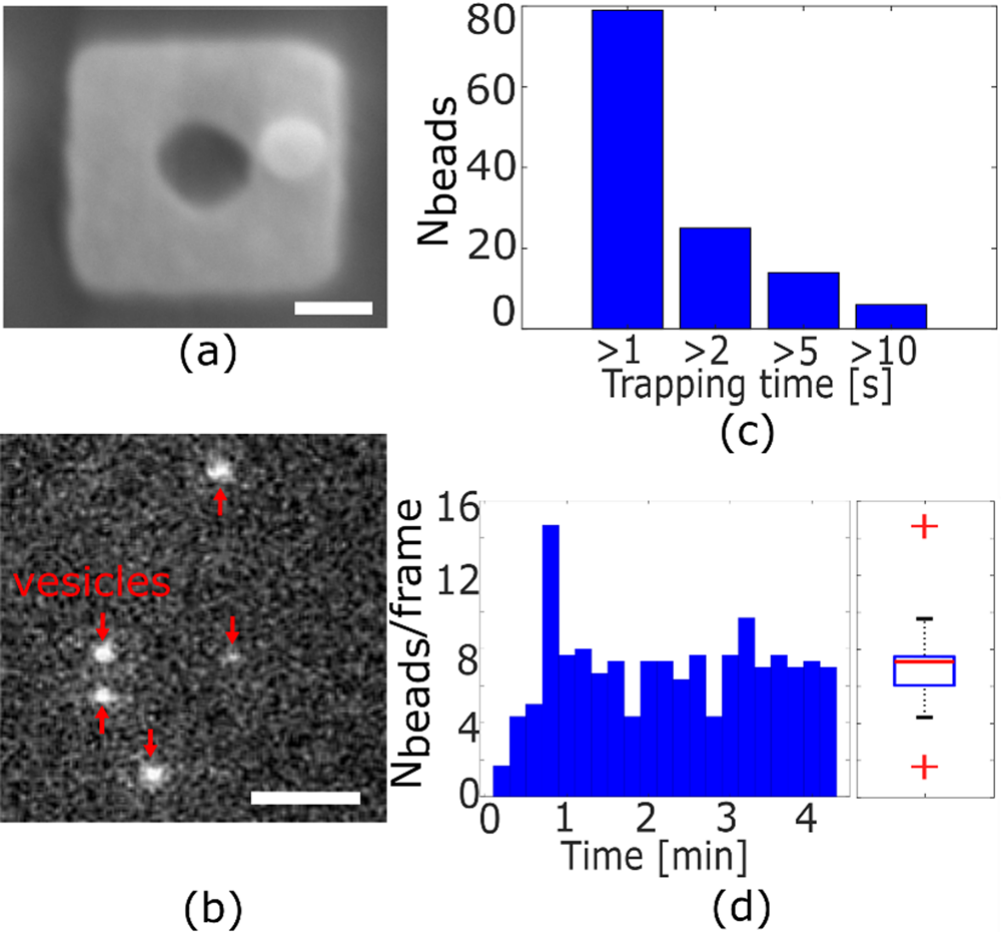
Trapping of unilamellar vesicles. (a) SEM micrograph of a unilamellar vesicle on a nanocuboid structure. The scale bar is 100 nm. (b) Multiplexed trapping of five unilamellar vesicles. The red arrows indicate the vesicle position. The scale bar is 2 μm (c) Number of vesicles trapped for *t* = 1 s, 2 s, 5 s, and 10 s and (d) vesicles trapped in parallel over an experiment of *t* = 5 min with *I* = 250 μW/μm^2^. The red lines in the box plots represent the median values of the displacement; the blue box and the black lines represent the 25th and 75th percentiles and the extreme values, while the red crosses are the outliers.

Finally, we validate the ability of the anapole array to trap biological particles. It is well understood that biological particles are more difficult to trap, because they offer a lower refractive index contrast than polystyrene beads; furthermore, they are more susceptible to photodamage and cannot tolerate high trapping intensities. Here, we use unilamellar lipid vesicles, because they represent an excellent model for viruses and exosomes.^23,24^ SEM micrographs and atomic force microscopy (AFM) measurements confirm a mean size of the vesicles of 101 ± 11 nm (Figure 5a, Supporting Information 10), which is very similar to the polystyrene beads used in the previous experiments. In order to aid experimental observation, the surface of the vesicles is labeled with 1,1′-dioctadecyl-3,3,3′,3′-tetramethylindocarbocyanine perchlorate (Dil).^41^ Dil is excited at 532 nm and emits in the 585–607 nm wavelength range. Because of the lower refractive index of the vesicles (*n* ∼ 1.45) compared to the polystyrene beads (*n* ∼ 1.57), we need to increase the intensity of the trapping laser and observe stable trapping for *I* ≥ 250 μW/μm^2^ (Figure 5b). We observe the trapping of *N* ∼ 80 particles over a time frame of *t* = 5 min, with tens of vesicles trapped for several seconds (*t* > 5 s) (Figure 5c). We also confirm the multiplexing capability by observing the parallel trapping of >10 vesicles (Figure 5d), with similar SIBA-induced high-density trapping area to that for the polystyrene particles (Figure 3c). We note that for a significantly higher intensity > 500 μW/μm^2^, damage and disruption of the vesicles occur, which confirms their susceptibility to photodamage. Being able to achieve low power trapping, as reported here, is therefore essential for biological experiments. Furthermore, the ability to minimize thermal variations is fundamental to minimize the probability of causing gel-to-liquid phase transitions, which could lead to variations in the biological properties of the vesicles.

## Conclusions

We studied the multiplexed trapping of nanoscale particles with a dielectric metasurface. The metasurface consists of an array of nanocuboids that support a coupled anapole state. The anapole state is critical for the trapping function because it supports a high-*Q*, strongly confined resonance; more importantly, it exhibits significant angular tolerance, which allows the excitation of the resonance with a focused laser beam. The laser beam is focused to an area of 30 μm in diameter, which allows for the parallel excitation of thousands of trapping sites with moderate power; we use a total power of ∼100 mW to achieve an intensity of 160 μW/μm^2^, which allows us to trap hundreds of 100 nm polystyrene beads in 10 min. We also validate the suitability of the anapole metasurface for trapping biological targets, here exemplified by unilamellar vesicles of 100 nm diameter, which we are able to trap with an optical power of 250 μW/μm^2^,^42^ i.e., well below the photodamage threshold. In this study, we have used labeled targets and a fluorescent-based detection scheme in order to achieve sufficiently high signal-to-noise ratio (SNR ∼ 5) and validate the multiplexing capability of the system. Furthermore, the use of labeled targets can provide higher specificity to analyze biological systems. However, we highlight the fact that the system is not limited to the use of fluorescence, but it can also be suitable for label-free detection schemes, based on the analysis of localized intensity variations to detect and characterize multiple targets, or also in combination with other label-free spectroscopic methods, such as Raman spectroscopy.^36,43,44^ The ability to trap a large number of objects on a limited time scale is extremely useful for many biological applications and for achieving conclusive statistics as well as understanding the heterogeneity of the biological system. The heterogeneity of response is highly relevant in many biomedical areas such as virology, oncology and immunology, disease progression, and response to drugs and treatments. We are confident that our demonstration of large-scale, low-power, multiplexed optical trapping will enable many opportunities for emerging studies in these areas.

## Experimental Section

### Numerical Simulations of the Metasurface

We carried out 3D FEM simulations (COMSOL Multiphysics) to design the dielectric metasurface and calculate the optical forces. We considered periodic boundary conditions around the unit cell in both directions to simulate an infinite size of the array. Perfectly matched layers (PML) are assumed at the top and bottom of the structure, preventing any light reflection that does not happen in a real scenario. For trapping simulations, we assumed the polystyrene bead and the vesicle as a 100 nm sphere with *n* = 1.57 and *n* = 1.45, respectively. The optical forces are calculated by integrating the Maxwell surface stress sensor on the bead surface. We have considered a multiphysics analysis to calculate the thermal effect in the chamber, using the near-field confinement as the main source of thermal heating (see Supporting Information 9).

### Dielectric Metasurface Fabrication

The nanocuboid array is realized in a-Si:H with a thickness of 100 nm on a glass substrate (Inseto UK). The sample is cleaned with a piranha solution (1:3 hydrogen peroxide–sulfuric acid) for 10 min and rinsed in deionized water, acetone, and isopropanol. The pattern of the nanocuboids is defined by e-beam lithography. We spin the positive resist ARP-9 (Allresist GmbH) at 4000 rpm for 60 s and bake at 180 °C for 5 min, obtaining a thickness of 200 nm. A 60 nm layer of AR-PC 5090 (Allresist GmbH) is spin-coated at 2000 rpm for 60 s and baked at 90 °C for 2 min for charge dissipation during the e-beam exposure. For the e-beam exposure (50 kV Voyager, Raith GmbH) we use a current of 140 pA and a dose of 130 μC/cm^2^. The sample is washed in deionized water for 2 min to remove the charge dissipation layer and then developed with xylene for 2 min, rinsed in isopropanol, and dried with nitrogen. The nanocuboid structure is finally transferred into the a-Si:H layer by reactive ion etching with a gas mixture of CHF_3_:SF_6_ = 14.5 sccm:12.5 sccm for 70 s with a voltage of 188 V and a chamber pressure of 0.4 mbar. Finally, we remove the remaining resist with 1165 solvent (Microchem) in a sonic bath at 50 °C for 10 min, followed by a rinse in acetone and isopropanol and a final drying step with nitrogen. Each sample includes several arrays of different periods. The period is tuned by 1 nm in order to overcome possible fabrication tolerances and always guarantee the perfect position of the resonance with respect to the trapping laser wavelength.

### Optical Characterization of the Metasurfaces

The metasurface is excited with a white light source (Leukos SM30). For the optical characterization of the metasurfaces (Figure 2a), the input light is focused by a Köhler lens (with 80 mm focal length) in the back focal plane of a 20× objective lens (Olympus PLN20X with NA = 0.4) to obtain a collimated beam to illuminate the nanocuboid array. The light reflected from the sample is then redirected by a beam splitter to a camera (Photometrics CoolSNAP DYNO) and to a spectrometer (Thorlabs, model CCS175) to reconstruct the optical spectrum. The spectrum collected from the metasurface is normalized to the signal measured by replacing the sample with a silver mirror, in order to quantify the resonance amplitude.

### Optical Setup for Trapping Measurements

The dielectric metasurface is excited with a laser diode at 787 nm (RLS/785NM-600MW) with a bandwidth of about 0.5 nm. We use the laser diode controller (RLS/MBL1500 v0.0) to regulate the power, and the temperature is kept at *T* = 16 °C. The input light is sent from the top and focused by a 20× objective lens (Olympus PLN20X with NA = 0.4) into the sample, facing downward. The trapping chamber is created with an O-ring (6 mm diameter and 0.3 mm thickness) used as a spacer between the sample and a glass coverslip. The chamber is filled with the solution containing the beads before sealing the chamber. For the imaging of the fluorescent beads, we have considered the best compromise between a large field of view and high imaging resolution. The fluorescence signal is excited by sending a single wavelength laser beam (Ventus 532) from the bottom of the chamber. The 532 nm laser beam is focused with a 20 mm lens in the back focal plane of a 50× objective (NIKON CFI TU PLAN EPI ELWD 50X with NA = 0.6), in order to illuminate the sample with a collimated light matching the excitation wavelength of the labeled beads and vesicles. Both beams have diameters of about 30 μm. The transmitted laser beam at λ = 787 nm and the reflected fluorescence signal are then collected by the 50× objective. The imaging resolution can be estimated by the well-known Abbe’s formula: *d* = λ/2NA with *d* being the minimum distance of two objects that can be resolved and lambda the operating wavelength. Assuming λ = 560 nm, corresponding to the peak of the emission wavelength of the labeled beads and vesicles, and NA = 0.6, we obtain a resolution of about 460 nm, which is very close to the periodicity used in the array (= 450 nm), confirming that it practically has the capability to distinguish two beads in adjacent traps. Three short-pass filters (FES0650, Thorlabs) are used to cancel out the signal at λ = 787 nm, while a long-pass filter (FELH0550, Thorlabs) is used to filter out the excitation wavelength at 532 nm. A bandpass filter with a center wavelength at λ = 559 nm and a bandwidth of 34 nm (MF559-34, Thorlabs) is used to collect the emitted fluorescence signal and minimize the background noise. The signal is collected by the camera (Photometrics CoolSNAP DYNO) with an integration time of 100 ms for the acquisition of each frame, controlled by the software μManager.

### Image Processing and Particle Tracking

The software Micro Manager is used to acquire the camera images, and the commercial software ImageJ is then used for the particle analysis. The software parameters are chosen in order to track particles that are trapped for at least 1 s, discarding all other beads. The number of spots and the corresponding trajectories are then saved in a text file, and a MATLAB script is then used to realize the plots reported in Figure 3 and Figure 4.

### SEM and AFM Characterization of Vesicles

Scanning electron microscopy (SEM) micrographs of vesicles nonspecifically bound to a silicon substrate are acquired with a JEOL JSM-7800F system operating at 5 kV. The solution with vesicles is manually spotted on the silicon substrate and allowed to evaporate. The vesicles left on the substrate are then covered with a 5 nm thick Pt/Pd layer, by sputtering, to avoid charging effects and possible damage to the vesicles during the image acquisition. Vesicle size is determined using ImageJ, and histograms are produced using 10 nm bin widths. AFM measurements (BioScope Resolve, Bruker) were taken to confirm the size and shape of the vesicles. A tapping modality with a frequency scan of 0.4 Hz and 256 lines with a resolution of about 5 nm is considered for the scanning of the individual vesicles. The images are processed with the software NanoScope Analysis 1.8.

### Sample Preparation for Polystyrene Bead Measurements

Fluorescence polystyrene beads (ThermoFisher, FluoSpheres carboxylate-modified microspheres F8800) with a mean size of 100 nm were used for the trapping experiments. The particles are stored at 4 °C prior to use. For the measurements, the nanobeads are diluted in deionized water with a concentration of 10 ng/mL and sonicated for 10 min. We introduced 0.1% Tween-20 in the solution in order to avoid clusters and work with individual beads. A 5 μL volume is manually pipetted on the dielectric array, and the chamber is sealed with a glass coverslip, using an O-ring (RS components) as a spacer layer to define the thickness (∼300 μm) of the trapping chamber.

### Preparation of 100-nm-Sized Vesicles

1-Palmitoyl-2-oleoyl-*sn*-glycero-3-phospho-l-serine (POPS) lipids in chloroform were purchased from Avanti Polar Lipids, stored at −20 °C prior to use, and used without any additional purification. The lipophilic membrane dye Dil (λ_ex_ = 550 nm, λ_em_ = 565 nm) was purchased from ThermoFisher Scientific and stored at 4 °C prior to use. Unilamellar vesicles of 100 nm size composed of 0.4 mol % Dil and 96.6% POPS were prepared using the extrusion method.^45^ Briefly, lipids and Dil were mixed in chloroform before the solvent was evaporated by the initial application of gentle nitrogen flow and subsequent desiccation under vacuum for 5 h.^45^ The dried lipid film was then resuspended in 50 mM Tris buffer adjusted to pH 8.0, mixed well by vortex, and extruded at least 21 times through a polycarbonate membrane filter with a size cutoff of 100 nm using a mini-extruder (Avanti Polar Lipids). Particle sizes were confirmed by dynamic light scattering using a Zetasizer mV molecular size detector (Malvern Instruments Ltd., UK) and with SEM images and AFM.

## Data Availability

The data that support the findings of this study and all codes produced during this research are available from the corresponding author at reasonable request.

## Supporting Information Available

Anapole state in the nanocuboid array configuration; energy enhancement in the nanocuboid structure at the anapole state; comparison of the energy confinement between the anapole state and the guided mode resonance; angle tolerance of the anapole state; 2D force distribution on a 100 nm dielectric bead (*n* = 1.45); distribution of trapped particles for different power values; optical forces exerted on 100 nm polystyrene beads; thermophoresis with high power values; thermal analysis of the anapole state; vesicle characterization with SEM and AFM; multiplexed trapping of 100 nm polystyrene beads; nanoparticle trapping and release by controlling the input power (PDF)
Supporting Movie 1: Multiplexed trapping of 100 nm polystyrene beads (AVI)
Supporting Movie 2: Nanoparticle trapping and release by controlling the input power (AVI)

## Supplementary Material

nn3c03100_si_001.pdf

nn3c03100_si_002.avi

nn3c03100_si_003.avi
