# Supplementary material for: Multiplexed Near-Field Optical Trapping Exploiting Anapole States
Source: ACS Nano. 2023 Aug 21;17(17):16695–702. doi: 10.1021/acsnano.3c03100 (PMC10510711; doi:10.1021/acsnano.3c03100)
Supplement: Supplementary file 1 — nn3c03100_si_001.pdf [file nn3c03100_si_001.pdf]

SUPPORTING INFORMATION

Multiplexed near-field optical trapping exploiting anapole states

Donato Conteduca[1], Giuseppe Brunetti[2], Isabel Barth[1], Steven D. Quinn[1,3], Caterina Ciminelli[2], Thomas F. Krauss[1,3]

[1]School of Physics, Engineering and Technology, University of York, Heslington, York YO10 5DD, United Kingdom

[2]Optoelectronics Laboratory, Politecnico di Bari, 70125 Bari, Italy.

[3]York Biomedical Research Institute, University of York, Heslington, York YO10 5DD, United Kingdom

Supporting Information 1. Anapole state in the nanocuboid array configuration

Supporting Information 2. Energy enhancement in the nanocuboid structure at the anapole state

Supporting Information 3. Comparison of the energy confinement between the anapole state and the guided mode resonance.

Supporting Information 4. Angle tolerance of the anapole state

Supporting Information 5. 2D Force distribution on 100nm dielectric bead (n=1.45)

Supporting Information 6. Distribution of trapped particles for different power values

Supporting Information 7. Optical forces exerted on 100 nm polystyrene beads

Supporting Information 8. Thermophoresis with high power values

Supporting Information 9. Thermal analysis of the anapole state

Supporting Information 10. Vesicles characterization with SEM and AFM

Supporting Movie 1. Multiplexed trapping of 100 nm polystyrene beads

Supporting Movie 2. Nanoparticles trapping and release by controlling the input power

Supporting Information 1. Anapole state in the nanocuboid array configuration

The dielectric metasurface realised with a nanocuboid array is designed to support an anapole mode at λ ~ 786 nm. An anapole state is a non-radiative resonance that can support a very high Q-factor and strong near-field confinement, and both properties are fundamental to enhance the trapping efficiency. An anapole resonance is obtained when the cross-sections of the electric ($Csca_p$) and toroidal dipoles ($Csca_t$) have the same intensity. The hollow core in the nanocuboid structure allows to control both the resonance position and the mode leaking outside the unit cell, so determining the resonance amplitude and Q-factor together with the energy confinement. A more detailed analysis on anapole resonances can be found in [22,23]. We have simulated the single unit-cell behaviour with 3D FEM simulations, assuming periodic boundary conditions in both directions. The electromagnetic simulation allows the decomposition of the electric and magnetic fields in the Cartesian multipole moments, in order to define the anapole position. The crossover of $Csca_p$ and $Csca_t$ is verified at λ_R ~ 786 nm (Figure S1a), generating a strong resonant response. As expected, the anapole state is characterized by a solenoidal electric field flow in-plane of the nanocuboid structure, while the toroidal magnetic field circulates out-of-plane of the unit-cell (Figure S1b and S1c). This behaviour is responsible for the energy enhancement by a factor 400 at the resonance, mainly confined in the hollow core.

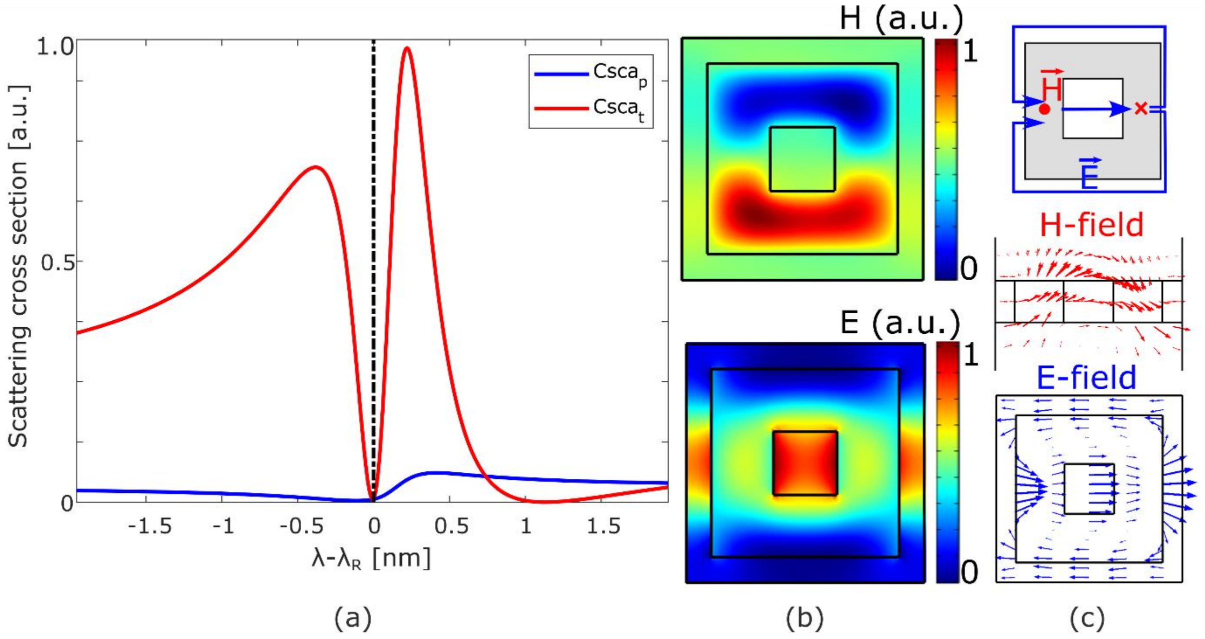

Figure S1. Study of the anapole state. *(a) Dielectric (blue curve) and toroidal (red curve) cross-sections, respectively $Csca_p$ and $Csca_t$, in the unit-cell of the nanocuboid array calculated with 3D FEM method. The crossover of $Csca_p$ and $Csca_t$ is verified at 786nm, where the dielectric metasurface supports the resonance condition. (b) Magnetic (top) and electric (bottom) field distribution in the nanocuboid structure at the resonance. (c) In-plane distribution of the E-field (blue curve) and out-of-plane circulation of the H-field (red curve) in the unit-cell.*

Supporting Information 2. Energy enhancement in the nanocuboid structure at the anapole state

We have compared with 3D FEM simulations the energy confinement in the nanocuboid structure at the resonance in the anapole state (ON-res) at $\lambda = 786$ nm and far from the resonance (OFF-res, $\lambda = 806$ nm). The numerical results confirm a field enhancement up to $E_{ON\text{-}res}/E_{OFF\text{-}res} > 20$ together with a much better localisation of the field in the hollow core of the nanocuboid (Figure S2), demonstrating the advantage offered by resonant structures to enhance the gradient field and, therefore, to maximise trapping strength.

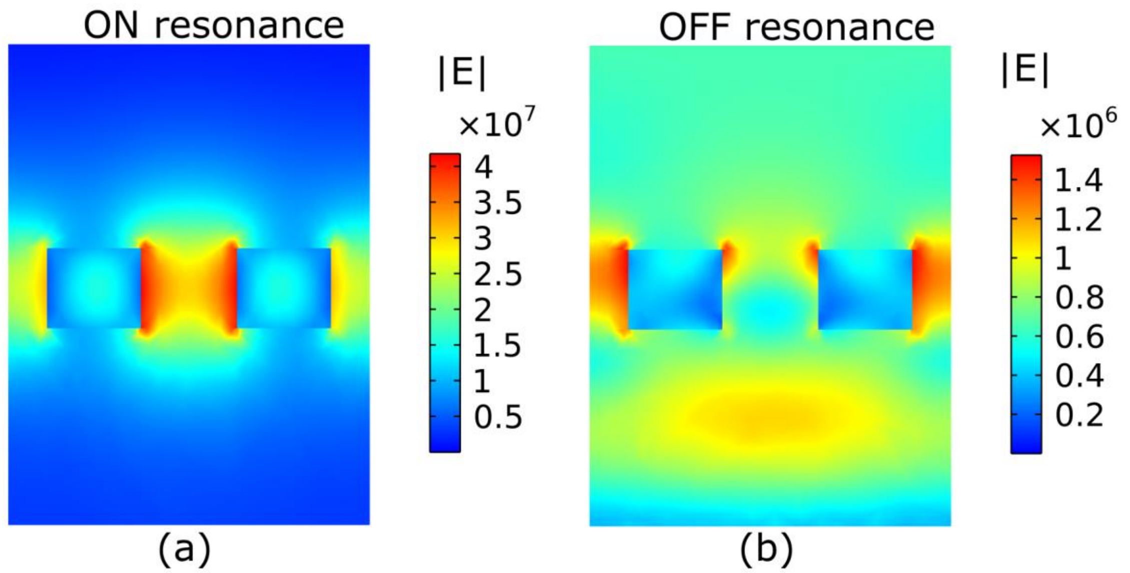

Figure S2. *Field confinement (a) at the resonance in the anapole state (λ = 786nm) and (b) far from the resonance (λ = 806nm).*

Supporting Information 3. Comparison of the energy confinement between the anapole state and the guided mode resonance.

The nanocuboid structure supports two resonances, the anapole state at λ = 786nm and the guided mode resonance at λ = 745.4 nm. We have considered the anapole state for trapping experiments because it presents stronger energy confinement (Figure S3), up to one order of magnitude higher than the guided mode resonance, together with a better field localisation in the trapping site and more angular tolerance, which are necessary to maximise the trapping strength.

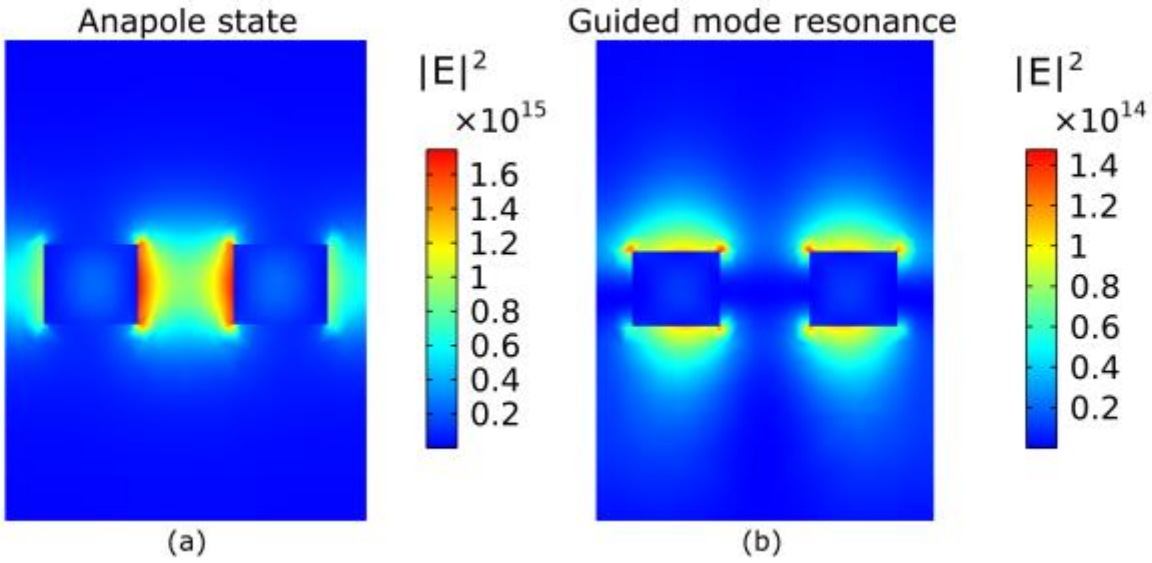

Figure S3. Cross-section of the energy confinement in the nanocuboid structure (a) in the anapole state and (b) guided mode resonance.

Supporting Information 4. Angle tolerance of the anapole state

We have studied the angle tolerance of the anapole mode with numerical simulations, assuming an incident plane wave for different values of the polar angle θ and an azimuthal angle Φ, to simulate the effect of a focused beam on both modes. We have considered an angle ranging from 0° (perpendicular illumination) to 20° for both θ and Φ (Figure S2a). The anapole mode presents a much stronger angle tolerance than the guided mode resonance, as confirmed by experimental results (Figure 2c). In particular, we have calculated the resonance shift and resonance properties for both modes for the cases (θ_i,Φ_i) with i = 0°,3°,6°,9° (Figure S4b). The resonance dependence on the angle of incidence can be usually approximated by a quadratic form ($\lambda_R(\theta) = \lambda_R(\theta=0°) + a\theta^2$) [S1], where a expresses the tolerance of the mode to the angle of incidence (lower values a correspond to a better angular tolerance). We have calculated that the anapole mode is described by a value of a that is lower by a factor 7 compared to the other resonant mode. Such behaviour is evident by the comparison of the spectra for different angles of incidence. For example, with θ = Φ = 9° we obtain a resonance shift of the anapole mode within 2 nm, smaller than the experimental bandwidth, while the other mode shows a shift of almost 60 nm (Figure S4c).

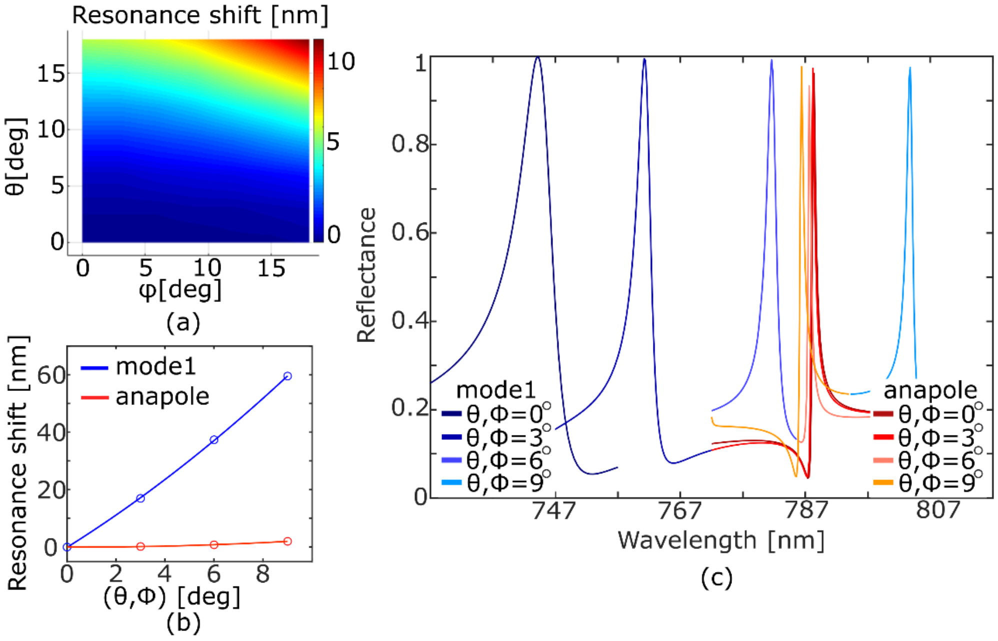

Figure S4. Numerical simulation of the angle tolerance of the anapole mode*. (a) 3D FEM simulation of the resonance shift of the anapole mode as a function of the azimuthal angle Φ and the polar angle θ. (b) Resonance shift calculated for (θ_i,Φ_i) with i = 0°,3°,6°,9° for both the anapole state and the other resonant mode (mode 1). The solid lines represent a quadratic fit. (c) Resonance spectra of the anapole mode (red curves) and mode 1 (blue curves) for (θ_i,Φ_i) with i = 0°,3°,6°,9°.*

(S1) Arruda, G.S., Conteduca, D., Barth, I., Wang, Y., Krauss, T.F., Martins, E.R., "Perturbation approach to improve the angular tolerance of high-Q resonances in metasurfaces", Optics Letters, **47**(23), pp. 6133-6136, 2022.

Supporting Information 5. 2D Force distribution on 100nm dielectric bead (n=1.45)

We have calculated the forces exerted on 100nm bead with n = 1.45 and I = 1 mW/µm^2. The force values were evaluated in the area within a single unit cell with the bead place above the nanocuboid surface (centre of the bead is 50 nm above the a-Si:H surface).

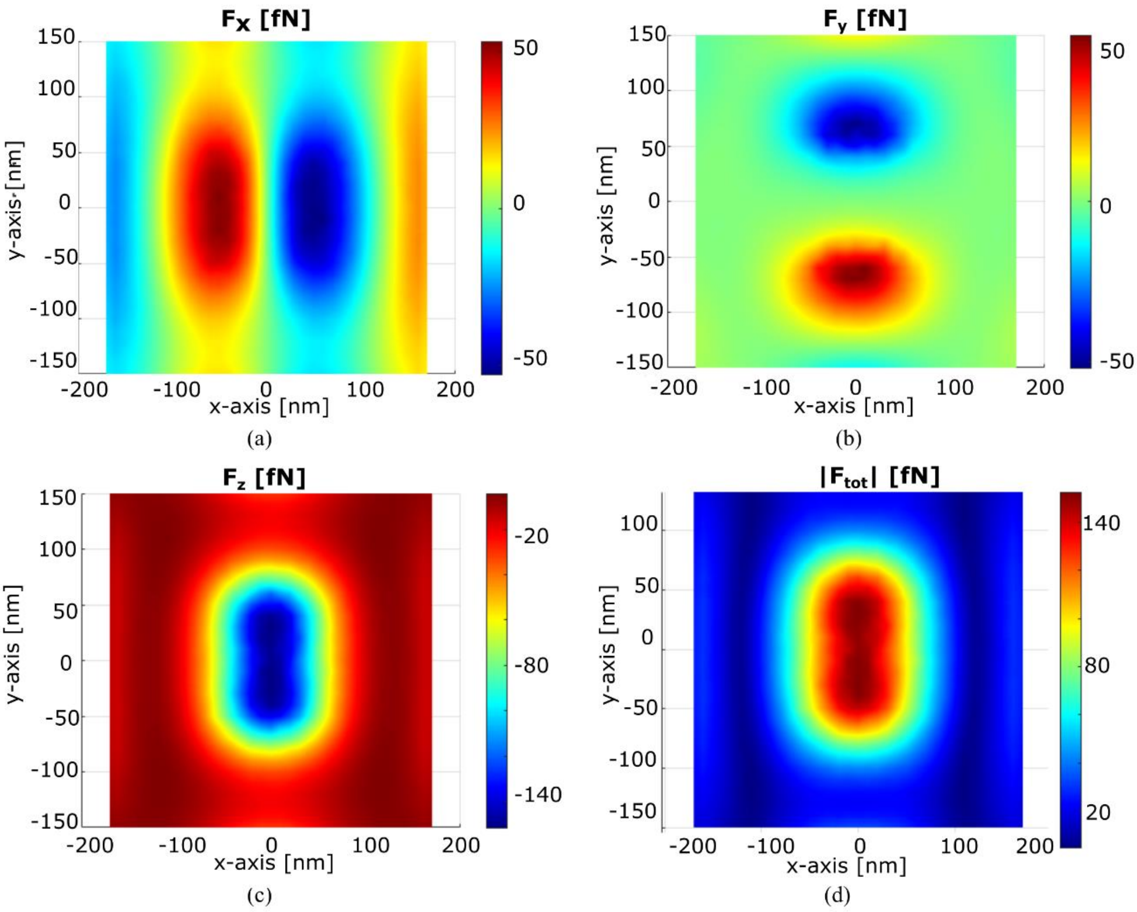

Figure S5. Force distribution with I = 1 mW/µm^2 for 100nm beads (n = 1.45) calculated on the plane of the nanocuboid surface along (a) x-axis, (b) y-axis, (c) z-axis and (d) absolute value of the total force.

Supporting Information 6. Distribution of trapped particles for different power values

We have evaluated the distribution of trapped beads for different values of input power, specifically I = 160 µW/µm^2 and I = 550 µW/µm^2. We track the position of 5 different 100 nm polystyrene particles trapped for at least 10 seconds for each power value (Figure S6a). We observe that for both values of power, the mean displacement of the beads is within the unit-cell, confirming the stable trapping condition in the array (Figure S6b). However, for I = 160 µW/µm^2 the beads can hop between adjacent unit-cells (Figure S6a) during the time they are trapped in the array. On the contrary, the higher trapping stability obtained with higher power values (I = 550 µW/µm^2) allows to localise the trapped beads within the single unit-cell (>90% of the total trapping time of each bead), confirmed by a mean displacement <100 nm.

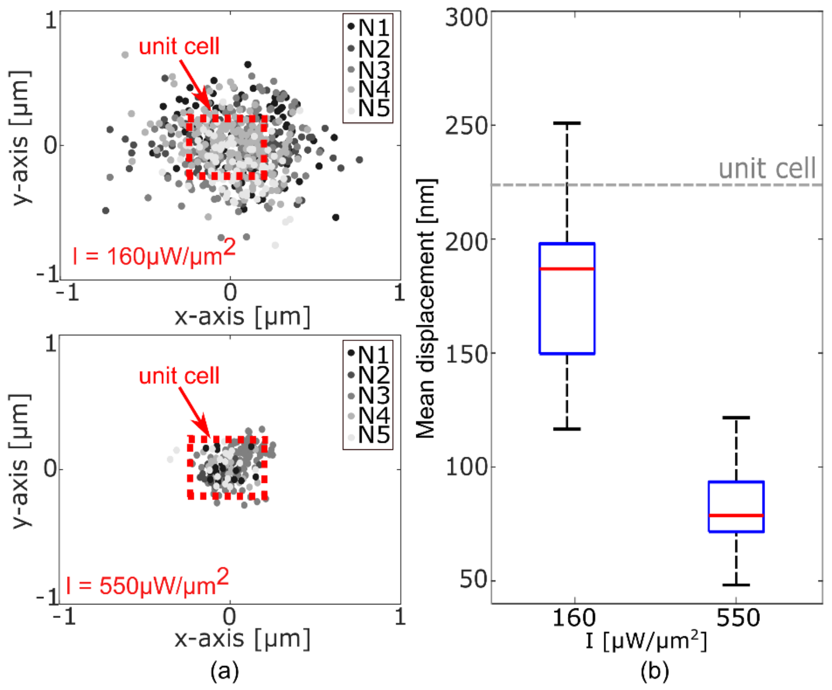

Figure S6. (a) Distribution of 5 polystyrene beads trapped for $t_{trap} \geq 10s$ with I = 160µW/µm^2 (top) and I = 550µW/µm^2 (bottom). (b) Mean displacement of the trapped beads. The red lines in the box plots represent the median values of the displacement; the blue box and the black lines represent the 25th and 75th percentiles and the extreme values of the mean position of 5 beads, respectively.

Supporting Information 7. Optical forces exerted on 100 nm polystyrene beads

We have calculated the optical forces exerted on a 100 nm polystyrene bead by applying the Maxwell Stress Tensor method, as described in the Methods section. The optical forces are higher compared to the values obtained with a bead with n = 1.45, due to the higher refractive index of the polystyrene bead (n = 1.57) (Figure S7a). Assuming an input power of 300 μW/μm^2 the maximum value of the attractive force is 2.4 pN. From a direct comparison with the bead with n = 1.45, for which a stability $S_{n = 1.45} = 10$ is calculated with such power value, this case of study with polystyrene beads shows an optical stability $S_{n = 1.57} = 13.8$, with an improvement of almost 40% (Figure S7b).

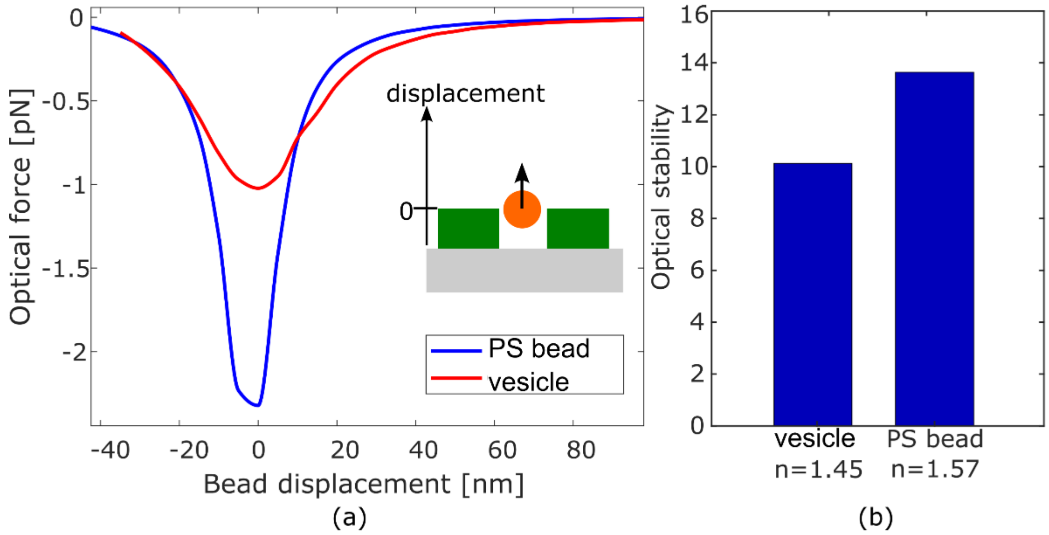

Figure S7. Evaluation of optical forces and optical stability. *(a) Comparison of the optical forces exerted on 100nm beads with n = 1.45 (vesicles) and n = 1.57 (polystyrene) with I = 300 μW/μm^2 and (b) correspondent optical stability.*

Supporting Information 8. Thermophoresis with high power values

Thermophoretic action is observed in the trapping chamber with high input power. The thermophoresis is originated from thermal gradients and affects the particles' motion by drifting them from hot areas to colder ones. Assuming a Gaussian distribution of the power for the laser beam, we expect the power, and therefore the energy confinement in the array, to decrease from the centre of the beam to the outer part. In this condition, we generate a negative thermal gradient with the beads pushed away with a velocity drift proportional to the thermal gradient. We have experimentally observed the thermophoretic action when the input power exerted on the array for trapping of 100 nm polystyrene beads overcomes 800 $\mu W/\mu m^2$. Figure S8 shows a large accumulation of beads outside the laser beam, exactly where the temperature rise is expected to be minimum. Thermophoresis action has been often used to assist optical trapping, but for this study of multiplexed trapping the thermophoresis is counterproductive because the strong localisation of beads in a small area causes loss of information about individual targets, as confirmed by the presence of multiple and large bead clusters (Figure S8).

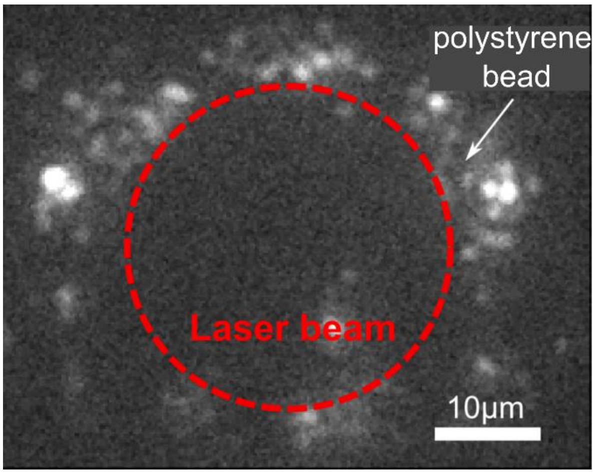

***Figure S8. Thermophoresis in the trapping chamber**. Particle localisation due to the thermophoretic action observed in the trapping chamber with $I > 800\ \mu W/\mu m^2$, with the beads pushed away from the laser beam and then localised in the outer area.*

Supporting Information 9. Thermal analysis of the anapole state

We have considered a Multiphysics analysis to calculate the field confinement and the temperature distribution in the nanocuboid array. For the optical simulation, we have initially considered a plane wave in input with periodic boundary conditions, in order to simulate an infinite array supporting a strong resonance. The periodic boundary conditions, considered only for the optical analysis and not in the thermal one, are placed around an area of a 10x10 array. Although the periodic boundary conditions would provide the same resonant behaviour even considering a single unit-cell, however, the choice of a larger array in the simulation of at least 10 unit-cells is very important for the thermal analysis.
The materials properties, mainly the absorption losses, and the field confinement are responsible for the thermal heating. Therefore, the electromagnetic confinement obtained at the resonance is considered as the main source (initial solution) for the thermal analysis in the nanocuboid structures. The computational volume chosen for the thermal analysis is 100 times larger in each direction than the initial array area, used for the optical simulation. After solving the optical analysis, we remove the periodic boundary conditions in order to simulate the heating dissipation in a realistic scenario. The choice to simulate an array of at least 10 unit-cells allows to avoid the presence of a "single hot-spot" in the thermal analysis, that we would have by considering a single unit-cell instead, so underestimating the maximum temperature increase.

We have calculated a temperature increase ΔT ~ 2.9 K (Figure S9), which corresponds to negligible thermophoretic effects for such power values, as demonstrated experimentally with the trapping of multiple beads. The limited thermal effect in the array is mainly related to the low values of optical absorption for the a-Si:H, which represents the main advantage of dielectric materials for near-field trapping applications.

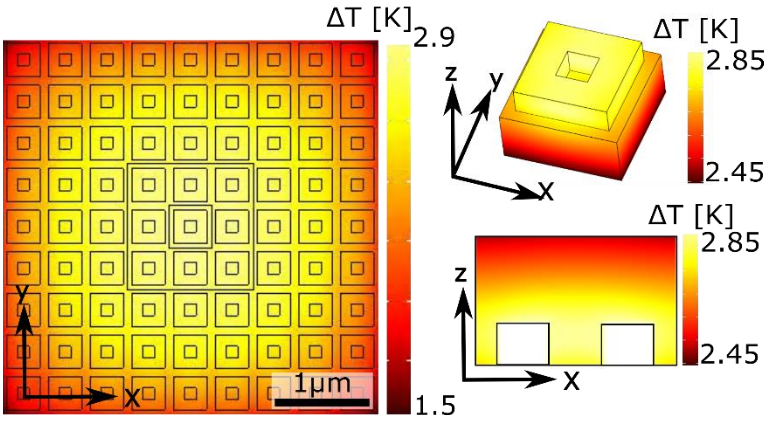

Figure S9. *Thermal analysis of the array on resonance for an input power of 160 μW/μm^2 (left), with the temperature distribution in a single unit cell and in the cross-section shown in the insets.*

Supporting Information 10. Vesicles characterization with SEM and AFM

The 100 nm-size unilamellar vesicles are spiked in deionised water and the solution is manually spotted on the silicon substrate. The preparation of the sample for SEM micrographs acquisition is described in detail in the Methods section. The acquired images (Figure S10a and S10b) are processed in ImageJ to calculate the size of the vesicles. We have considered N = 50 vesicles for a statistically relevant analysis. We have measured a mean value of 101nm ±11nm (Figure S10d). We have discarded in the analysis all vesicles larger than 200nm, corresponding to possible clusters of multiple vesicles. AFM images taken with BioScope Resolve (Bruker) confirm the particles size previously obtained with SEM micrograph (Figure S10b). The analysis confirms that 94% of vesicles present a diameter < 115 nm (Figure S10c), which is the necessary condition to fit within the hollow core of the nanocuboid structure, in order to exert the strongest trapping action.

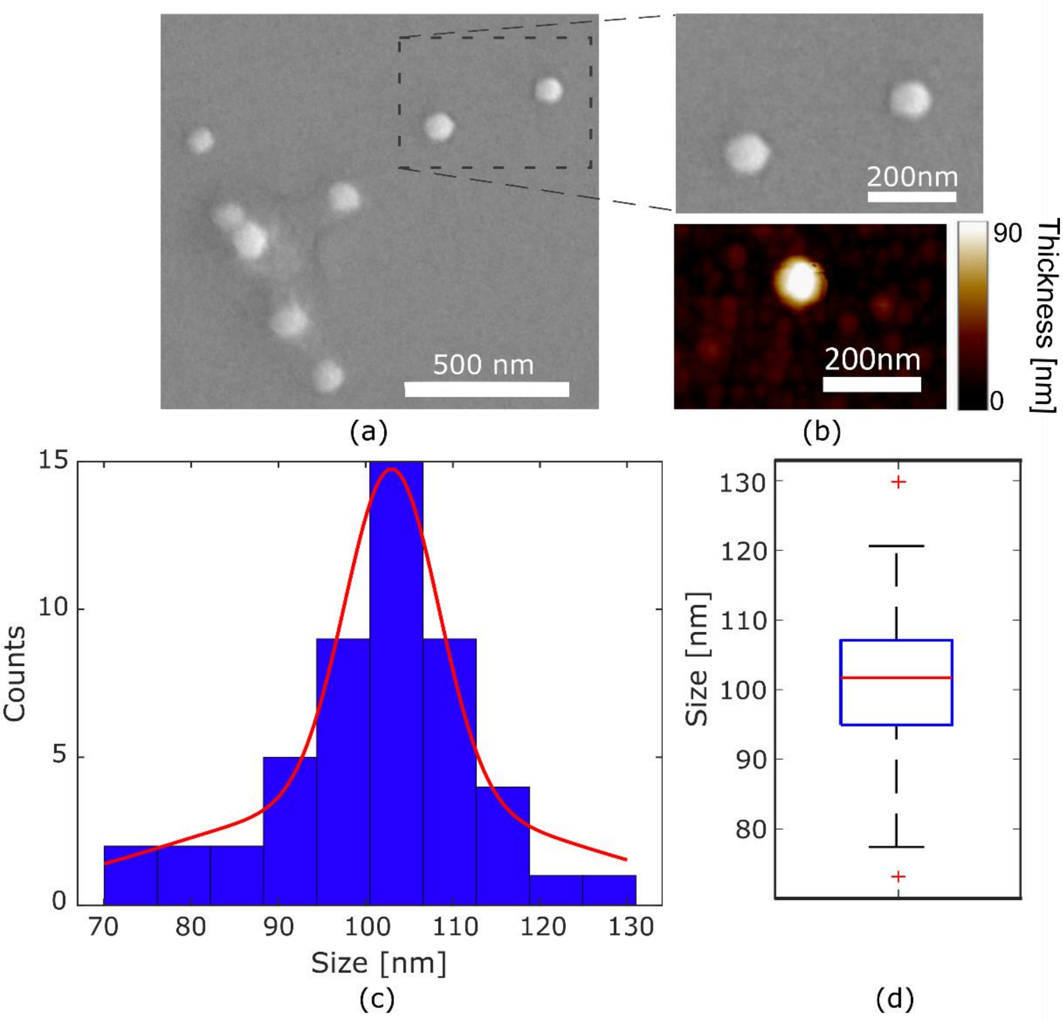

Figure S10. Vesicles characterization. *(a) SEM micrograph with N = 8 vesicles in the field of view and (b) SEM magnification (top) and AFM analysis (bottom) of individual vesicles. (c) Statistical study with Gaussian distribution (red curve) on N = 50 vesicles confirming a (d) mean size of 101nm and 94% of vesicles with a diameter < 115nm.*
